# A preliminary study on brain functional magnetic resonance imaging in migraine patients with visual aura using visual stimulation

**DOI:** 10.3389/fneur.2026.1742630

**Published:** 2026-07-08

**Authors:** Jinyao Li, Mu Lan, Xin Yang, Xiaoming Wang, Quansheng Liu, Xinyi Wang

**Affiliations:** 1 Department of Neurology, Affiliated Hospital of North Sichuan Medical College, Nanchong, China; 2 Interventional Medicine Center, Affiliated Hospital of North Sichuan Medical College, Nanchong, China; 3 Department of Rehabilitation Medicine, Affiliated Hospital of North Sichuan Medical College, Nanchong, China; 4 Dazhou Dachuan District People’s Hospital (Dazhou Third People’s Hospital), Dazhou, China

**Keywords:** brain networks, magnetic resonance imaging, migraine, visual aura, visual stimulation

## Abstract

**Background and Objective:**

Migraine is characterized by high prevalence, recurrent attacks, and poor response to pharmacological interventions, which can severely impair patients’ quality of life. The complex and diverse aura symptoms in its clinical presentation further increase the risk of misdiagnosis and mismanagement. Moreover, the pathogenesis of this disorder remains incompletely understood. This study employed a visual stimulation paradigm combined with blood oxygenation level-dependent functional magnetic resonance imaging (BOLD-fMRI) to simultaneously monitor brain functional activity and neuronal dynamics in patients with visual aura migraine (VaM) and migraine without aura (MwoA). The aim was to identify specific brain network features associated with visual aura in VaM, thereby providing new insights into its pathological mechanisms and early differential diagnosis.

**Methods:**

From May 2023 to May 2024, 21 patients meeting the diagnostic criteria for VaM and excluding related ocular fundus and optic nerve pathologies during the interictal period (VaM group), 21 patients meeting the criteria for MwoA during the interictal period (MwoA group), and 21 gender- and age-matched healthy controls (HC group) were recruited from the Department of Neurology, Affiliated Hospital of North Sichuan Medical College. All participants underwent visual stimulation using an 8 Hz flickering checkerboard paradigm concurrent with task-state fMRI scanning and image acquisition. The primary outcome was the comparison of brain activation differences among the three groups and between each pair of groups using one-way analysis of variance (ANOVA) and post-hoc multiple comparison analyses. Secondary outcomes included within-group brain activation analysis for the VaM, MwoA, and HC groups using one-sample t-tests.

**Results:**

Brain regions showing differential activation among the VaM, MwoA, and HC groups included: right cerebellar lobule IX, left middle temporal gyrus, left orbital part of the inferior frontal gyrus, left triangular part of the inferior frontal gyrus, left opercular part of the inferior frontal gyrus, left temporal pole: superior temporal gyrus, left medial superior frontal gyrus, left anterior cingulate gyrus, left middle cingulate gyrus, right middle cingulate gyrus, right middle occipital gyrus, left middle occipital gyrus, left superior occipital gyrus, left middle temporal gyrus, right angular gyrus, left precuneus, right precuneus, and left cuneus (*p <* 0.05). Pairwise comparisons revealed: Compared to the HC group, the VaM group exhibited stronger activation in the left precuneus (*p <* 0.005). Compared to the HC group, the MwoA group showed stronger activation in the right middle occipital gyrus (*p <* 0.005) and left middle occipital gyrus (*p <* 0.05). Compared to the MwoA group, the VaM group demonstrated stronger activation in the right angular gyrus (*p <* 0.05), left cuneus (*p <* 0.0001), right middle cingulate gyrus (*p <* 0.05), and left precuneus (*p <* 0.05). Conversely, the VaM group showed weaker activation than the MwoA group in the right middle occipital gyrus (*p* < 0.05).

**Conclusion:**

The results indicate abnormalities in visual-related brain networks in both patient groups, suggesting a central mechanism underlying visual aura generation in VaM. Abnormal activation in regions such as the cuneus, precuneus, and superior occipital gyrus may serve as preliminary indicators that warrant further investigation into visual aura mechanisms, while changes in the middle cingulate gyrus and angular gyrus may be associated with cognitive-emotional aspects of migraine, although this remains exploratory.

## Introduction

Migraine is a common primary headache disorder characterized by recurrent attacks and often a poor response to medication. Epidemiological studies report a global prevalence of 10–18%, with a prevalence of approximately 9.3% in China, and the trend is increasing (1–6). Approximately one-third of migraine patients experience aura; visual aura is the most common (about 90%), and some patients present with multiple concomitant aura symptoms (7–13). Because migraine aura symptoms are complex, misdiagnosis and inappropriate treatment can occur in clinical practice. Visual aura symptoms are typically transient, lasting about 15 min, and are often overshadowed by the subsequent headache and other associated symptoms. Nevertheless, some patients are severely affected by visual aura, leading to a marked decline in quality of life (14).

Functional magnetic resonance imaging (fMRI) is a non-invasive neuroimaging technique. BOLD-fMRI relies on the different magnetic properties of deoxygenated and oxygenated hemoglobin. When neuronal activity increases, cerebral blood flow in the activated area rises significantly, decreasing deoxygenated hemoglobin concentration and increasing the T2-weighted signal (15). The pathogenesis of migraine remains unclear; several hypotheses have been proposed, including cortical spreading depression (CSD), vasodilation, trigeminovascular activation, and neurogenic inflammation (16–19). In 1947, Leao observed an inhibitory wave in animal experiments that spread centrifugally from the stimulation electrode at a rate of 2–3 mm/min, a phenomenon he termed CSD (20). CSD is now widely accepted as a key mechanism in migraine pathogenesis and is considered the basis of visual aura. Previous work has shown that occipital cortex blood flow changes in VaM patients correspond to the retinotopy of visual perception, with attenuated blood flow signals and hemodynamic responses after identical retinal progression (21). Hougaard et al. (22) found selectively increased BOLD responses in the inferior parietal lobule, inferior frontal gyrus, and superior parietal lobule of VaM patients during the interictal period. These findings suggest abnormal brain network organization in VaM. Thus, exploring the central mechanisms of migraine, particularly visual aura, is necessary.

Most previous research has focused on resting-state fMRI rather than task-state fMRI. In this study, we applied visual stimulation to patients with VaM and MwoA and used BOLD imaging to simultaneously monitor functional brain areas and corresponding neuronal activity changes. Our aim was to investigate the brain network characteristics associated with visual aura in VaM, hoping to provide clues for understanding its pathogenesis and facilitating early diagnosis or differential diagnosis.

## Methods

### Participants

A total of 42 migraine patients were recruited from the Department of Neurology, Affiliated Hospital of North Sichuan Medical College, between April 2023 and May 2024. This cohort comprised 21 patients with visual aura migraine (VaM group) and 21 with migraine without aura (MwoA group). In the same period, 21 healthy controls (HC group) matched for sex and age were recruited.

Inclusion criteria for the VaM and MwoA groups were: (1) diagnosis of VaM or MwoA according to the Chinese guidelines for the diagnosis and treatment of migraine (23); (2) age between 15 and 68 years; (3) right-handedness; (4) no abnormalities on neurological examination; (5) ability to cooperate with the fMRI examination and provision of written informed consent; (6) no contraindications to MRI; (7) absence of ophthalmological diseases (e.g., cataract, glaucoma, retinopathy) or any other condition that could potentially affect the study; (8) no intake of antiepileptic drugs, antipsychotics, or other relevant medications within 48 h before the examination.

Exclusion criteria were: (1) ocular diseases: refractive error (> ± 6.00 D or astigmatism), severe lens opacity, glaucoma, retinopathy, etc.; (2) diseases that might affect the visual system, such as diabetes mellitus or uncontrolled hypertension; (3) other headache types or neuropsychiatric disorders besides migraine (e.g., tension-type headache, cluster headache, multiple sclerosis, neuromyelitis optica spectrum disorders, cerebrovascular diseases, or depression); (4) inability to cooperate with and complete the experiment.

### Experimental apparatus

Visual stimulation was delivered using a Visual and Audio Stimulation System for fMRI (Jiexin (Shenzhen) Technology Co., Ltd.). The study was conducted at the 3.0 T MRI Center of the Affiliated Hospital of North Sichuan Medical College. A 3.0 T superconducting MR scanner (GE Healthcare, United States) was used for image acquisition, with signals received via a 32-channel phased-array head coil.

### Clinical data collection

All participants first underwent collection of demographic and general clinical data, including gender, age, years of education, disease duration, Visual Analogue Scale (VAS) score, and Mini-Mental State Examination (MMSE) score. The severity of migraine pain was assessed using the VAS, ranging from 0 to 10, with higher scores indicating more severe pain. Cognitive function was evaluated for all subjects using the MMSE (total score 30). The cut-off scores for cognitive impairment were defined as follows: <17 for illiterate individuals, <20 for those with elementary school education, <24 for those with junior high school or secondary specialized school education, and <27 for those with high school education or above.

### Visual stimulation paradigm

**Figure 1:**
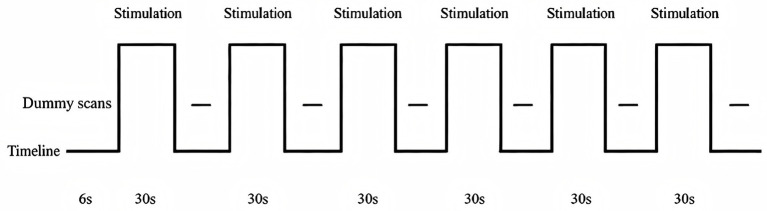
Schematic diagram of the visual stimulation paradigm. The first 6 s (2 volumes) are dummy scans (discarded initial volumes/pre-synchronization period).

Two experienced MRI technicians performed the fMRI scanning using a visual stimulation paradigm combined with an event-related design. Task-state fMRI scanning was synchronized with the presentation of a standardized flickering checkerboard paradigm (alternating at 8 Hz). The paradigm consisted of 6 blocks of 60 s each. Each block comprised 30 s of visual stimulation (flickering checkerboard) followed by 30 s of rest (fixation crosshair). The first 6 s of the scan (2 volumes, given a TR of 3,000 ms) served as dummy scans (pre-synchronization period) and were discarded before analysis to allow for signal stabilization. Stimulus presentation was synchronized with the scanner via an optical relay triggered by the radiofrequency pulse. Total acquisition time was 6 min and 6 s, yielding 120 volumes (TR = 3,000 ms). During the experiment, participants were instructed to fixate on a central crosshair on the screen, to minimize blinking, and to blink gently when necessary to avoid excessive head motion and saccadic eye movements. The specific design of the visual stimulation paradigm is detailed in Figure 1.

### Functional magnetic resonance imaging data acquisition

The scanning protocol commenced with conventional T1-weighted magnetization-prepared rapid acquisition gradient echo (T1 MP-RAGE), T2-weighted fluid-attenuated inversion recovery (T2 FLAIR), and T2-weighted imaging (T2WI) sequences. Following the conventional scans and upon confirmation of no intracranial structural abnormalities, resting-state fMRI (rs-fMRI) was performed to acquire baseline brain activity data for subsequent comparison with the visual stimulation task. Subsequently, the visual stimulation system was activated, and participants were stimulated according to the pre-designed paradigm while simultaneous task-state fMRI data were acquired to detect changes in brain activity following visual stimulation. All fMRI data were acquired using a gradient-echo echo planar imaging (EPI) sequence covering the whole brain. The total acquisition time for fMRI was 6 min and 6 s. The first 6 s of images were discarded to account for magnetic field instability at the beginning of the scan, resulting in a total of 120 time points being collected. Detailed MRI scanning parameters are provided in Table 1.

**Table 1:** MRI scanning parameters.

Sequence type	Pulse sequence	TR (ms)	TE (ms)	FA/RA (°)	Matrix	Slice thickness (mm)
T1 structural image	3D FSPGR BRAVO	8.3	3.2	12	256 × 256	1
T2-FLAIR	2D fluid-attenuated IR	6,416	140	111	192 × 160	6
T2WI	2D fast spin echo	9,332	93	140	256 × 256	5
fMRI	EPI BOLD	3,000	30	90	64 × 64	3.5

### Ethical approval

This study was approved by the Ethics Committee of the Affiliated Hospital of North Sichuan Medical College (approval no. 2024ER373-1). All participants provided voluntary informed consent after receiving a detailed explanation of the study procedures. For participants under 18 years of age (*n* = 2 in the VaM group, aged 15 and 17), written informed consent was obtained from their parents or legal guardians, and the minors themselves provided written assent. Furthermore, as stipulated in the protocol, no participants had taken antiepileptic drugs, antipsychotics, or other relevant medications within 48 h prior to the scan.

### Data processing and analysis

Statistical analyses were performed using SPSS software (version 26.0). The normality of continuous variables for demographic and basic clinical data was assessed. Data conforming to a normal distribution are presented as mean ± standard deviation (± s), while skewed data are presented as median (first quartile, third quartile) [M (Q1, Q3)]. Categorical data (gender) were compared among the three groups using the Chi-square test. Years of education and MMSE scores, which were skewed, were compared using the Kruskal-Wallis H test. Age, which was normally distributed, was compared using one-way analysis of variance (ANOVA). Disease duration (skewed) was compared between patient groups using the Mann–Whitney U test. The VAS score (normally distributed) was compared between patient groups using the independent samples t-test. All hypothesis tests were two-tailed. For the primary whole-brain fMRI analysis, a voxel-wise threshold of *p* < 0.05 (uncorrected) with a minimum cluster size of 8 contiguous voxels was applied. Given the exploratory nature of the study and the relatively modest sample size, this threshold prioritized sensitivity to detect potentially relevant brain regions while accepting a higher risk of type I errors. No *a priori* regions of interest (ROIs) were defined, making the whole-brain analysis inherently exploratory.

Imaging data from all participants were preprocessed using Statistical Parametric Mapping 12 (SPM12; http://www.fil.ion.ucl.ac.uk/spm) and MATLAB 2018b (MathWorks Inc.). The preprocessing pipeline included the following steps in order: 1. Slice timing correction: To ensure temporal consistency across slices, images were corrected to the middle slice acquisition time. 2. Head motion correction (realignment): All volumes were realigned to the first volume. Participants with maximum head motion exceeding 2 mm in translation or 2° in rotation in any direction were excluded. None of the 63 participants met this exclusion criterion. Motion parameters were used for quality control only and not entered as regressors in the first-level model. 3. Spatial normalization: Each participant’s structural image was coregistered to the functional images, and then normalized to the Montreal Neurological Institute (MNI) space using the SPM12 unified segmentation-normalization algorithm. Functional images were resampled to 3 × 3 × 3 mm^3^ voxels. 4. Spatial smoothing: A Gaussian kernel of 8 mm full-width at half-maximum (FWHM) was applied to increase signal-to-noise ratio and accommodate inter-individual anatomical variability (24). 5. Filtering and denoising: In addition to the default high-pass filter implemented within SPM12’s general linear model (with a cutoff of 128 s to remove low-frequency drift), we explicitly removed several sources of noise. Specifically, we regressed out linear trends (e.g., scanner temperature drift), head motion parameters (six rigid-body parameters obtained from realignment), whole-brain mean signal, and cerebrospinal fluid (CSF) signal. These steps were applied to reduce interference from non-neuronal fluctuations. No additional temporal filtering (e.g., dedicated band-pass filter) was applied beyond the default SPM12 modeling, as the task-related block design and the relatively short acquisition time made it less critical. For first-level analysis, a general linear model (GLM) was employed to model the hemodynamic response for each participant at different stimulus time points. To compare brain activation among the VaM, MwoA, and HC groups (all participants having passed ophthalmological exclusion criteria), a one-way analysis of variance (ANOVA) was performed at the second level. Post-hoc pairwise comparisons were then conducted using the Least Significant Difference (LSD) test to examine statistically significant differences in brain activation between each pair of groups. The statistical significance threshold was set at *p* < 0.05 (uncorrected). Furthermore, only clusters exceeding a size of 8 contiguous voxels were considered statistically meaningful to mitigate false positives.

## Results

### Demographic and clinical characteristics

The final study cohort consisted of 21 patients with VaM (17 females, 4 males; age range 15–68 years, mean ± SD 33.24 ± 3.39 years). Visual aura manifestations included phosphenes (*n* = 7), scintillating scotoma (*n* = 5), blind spots (*n* = 5), and visual field defects (*n* = 4). The MwoA group comprised 21 patients (13 females, 8 males; age 21–53 years, mean ± SD 29.67 ± 1.60 years). The HC group included 21 healthy subjects (16 females, 5 males, age 21–33 years, mean ± SD 28.10 ± 0.66 years). No significant differences were observed among the three groups in age, sex, or years of education (all *p* > 0.05). Similarly, no significant differences were found between the VaM and MwoA groups in VAS scores, MMSE scores, or disease duration (all *p* > 0.05). Notably, eight VaM patients with a longer disease course had experienced transient anxiety and depression during the interictal period. Detailed clinical information for all 63 participants is provided in Table 2.

**Table 2:** Statistical analysis of demographic and clinical data among the three groups.

Item	VaM group	MwoA group	HC group	*p* value
Number (*n*)	21	21	21	-
Female, *n* (%)	17(81.00)	13(61.90)	16(76.20)	0.351
Age (years)	33.24 ± 3.39	29.67 ± 1.60	28.10 ± 0.66	0.345
Years of education (years)	15(11.50–15.00)	15(15.00–17.00)	15(14.00–16.00)	0.640
Disease duration (years)	6(3.50–10.00)	8(5.50–10.00)	-	0.193
VAS score	5.86 ± 0.19	5.67 ± 0.24	-	0.567
MMSE score	30(30.00–30.00)	30(30.00–30.00)	30(30.00–30.00)	0.750

VaM, visual aura migraine; MwoA, migraine without aura; HC, healthy control; VAS, Visual Analog Scale; MMSE, Mini-Mental State Examination.

### Within-group activation analysis

Brain regions activated in the VaM group included: the middle occipital gyrus, inferior occipital gyrus, lingual gyrus, pericalcarine cortex, fusiform gyrus, cerebellar lobule VI, cerebellar tonsil 1, cuneus, putamen, insula, rolandic operculum, opercular part of the inferior frontal gyrus, triangular part of the inferior frontal gyrus, and supplementary motor area (*p* < 0.05).

The MwoA group showed activation in: cerebellar tonsil 1, cerebellar tonsil 2, cerebellar lobule VIIb, cerebellar lobule VIII, cerebellar lobule IX, superior temporal gyrus, middle temporal gyrus, inferior temporal gyrus, hippocampus, parahippocampal gyrus, fusiform gyrus, superior occipital gyrus, middle occipital gyrus, inferior occipital gyrus, lingual gyrus, pericalcarine cortex, cerebellar tonsil 1, cerebellar lobule VI, cuneus, precentral gyrus, postcentral gyrus, paracentral lobule, insula, rolandic operculum, supplementary motor area, opercular part of the inferior frontal gyrus, triangular part of the inferior frontal gyrus, putamen, supramarginal gyrus, middle frontal gyrus, angular gyrus, pallidum, orbital part of the inferior frontal gyrus, rolandic operculum, temporal pole: superior temporal gyrus, middle frontal gyrus, precuneus, middle cingulate gyrus, posterior cingulate gyrus, angular gyrus, caudate nucleus, and superior parietal gyrus (*p* < 0.05).

**Figure 2:**
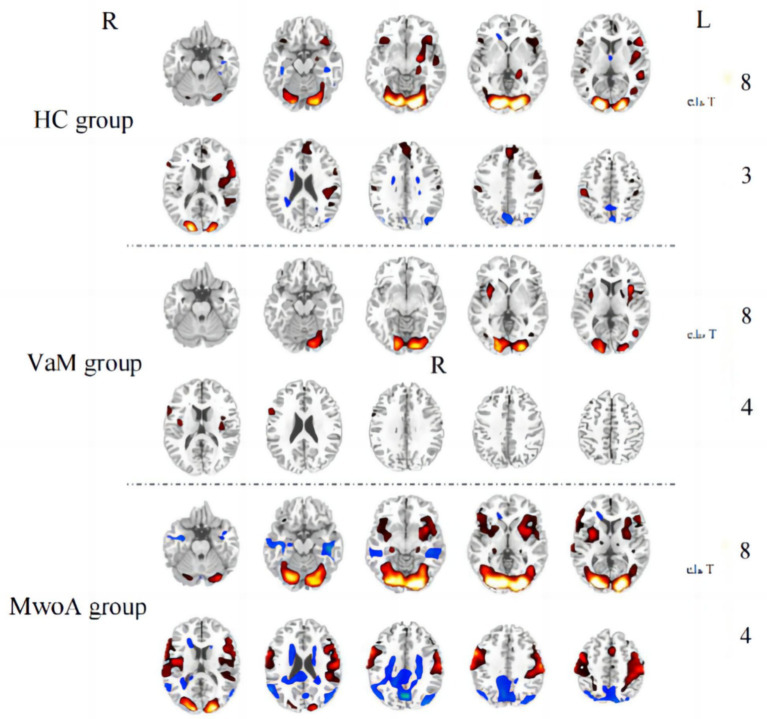
Distribution of activated brain regions in the VaM, MwoA, and HC groups. Yellow to blue color represents activation intensity from strong to weak; T-value: T value; R: right, L: left.

Activated regions in the HC group encompassed: cerebellar tonsil 1, cerebellar tonsil 2, cerebellar lobule VI, cerebellar lobule VIIb, cerebellar lobule VIII, lingual gyrus, pericalcarine cortex, fusiform gyrus, superior occipital gyrus, middle occipital gyrus, inferior occipital gyrus, orbital part of the inferior frontal gyrus, triangular part of the inferior frontal gyrus, opercular part of the inferior frontal gyrus, insula, hippocampus, parahippocampal gyrus, superior temporal gyrus, middle temporal gyrus, inferior temporal gyrus, rolandic operculum, precentral gyrus, postcentral gyrus, supramarginal gyrus, temporal pole: superior temporal gyrus, angular gyrus, medial superior frontal gyrus, middle cingulate gyrus, dorsolateral superior frontal gyrus, and precuneus (*p* < 0.05). The brain activation maps for the VaM, MwoA, and HC groups are detailed in Figure 2.

### Between-group activation analysis

**Figure 3:**
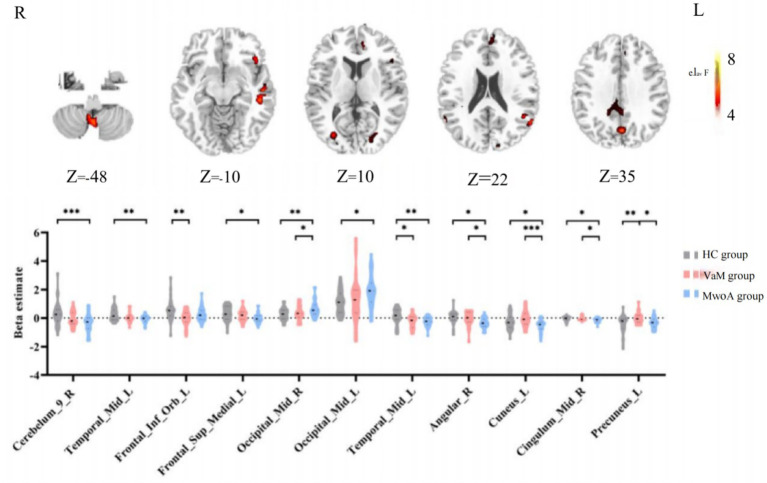
Comparative analysis of brain activation differences among the three groups. Yellow to blue indicates the strength of activation from strong to weak. ^*^p < 0.05, ^**^p < 0.005, ^***^p < 0.001.

One-way ANOVA revealed significant differences in brain activation among the VaM, MwoA, and HC groups in the following regions: right cerebellar lobule IX, left middle temporal gyrus, left orbital part of the inferior frontal gyrus, left triangular part of the inferior frontal gyrus, left opercular part of the inferior frontal gyrus, left temporal pole: superior temporal gyrus, left medial superior frontal gyrus, left anterior cingulate gyrus, right middle cingulate gyrus, left middle cingulate gyrus, right middle occipital gyrus, left middle occipital gyrus, left superior occipital gyrus, left middle temporal gyrus, right angular gyrus, left precuneus, right precuneus, and left cuneus (*p* < 0.05, uncorrected). The differences in brain activation among the three groups are detailed in Figure 3 and Table 3.

**Table 3:** Results of ANOVA for brain activation among the three groups.

Activated brain region	ROI	MNI coordinates			T-value	Cluster size (voxels)
		X	Y	Z		
Cerebellum_9_R	106	3	−54	−48	6.723	22
Temporal_Mid_L	85	−54	−18	−12	5.946	64
Frontal_Inf_Orb_L	15	−42	24	−15	5.918	49
Frontal_Inf_Tri_L	13					17
Frontal_Inf_Oper_L	11					10
Temporal_Pole_Sup_L	83					9
Frontal_Sup_Medial_L	23	−6	57	18	4.699	32
Cingulate_Ant_L	31					21
Occipital_Mid_R	52	30	−78	9	5.486	8
Occipital_Mid_L	51	−21	−87	9	4.222	14
Occipital_Sup_L	49					11
Temporal_Mid_L	85	−57	−60	21	5.733	20
Angular_R	66	57	−57	24	5.910	25
Precuneus_L	67	0	−69	30	7.253	24
Cuneus_L	45					16
Precuneus_R	68					10
Cingulate_Mid_R	34	18	−48	30	4.953	35
Cingulate_Mid_L	33					15
Precuneus_L	67					12
Precuneus_L	67	−9	−54	72	5.567	21

Post-hoc analysis using the Least Significant Difference (LSD) test showed that, compared to the HC group, the VaM group exhibited significantly stronger activation in the left precuneus (^*^p < 0.05, ^**^p < 0.005, ^***^p < 0.001). The differences in brain activation between the VaM and HC groups are illustrated in Figure 3.

Compared to the HC group, the MwoA group demonstrated stronger activation in the right middle occipital gyrus and left middle occipital gyrus (^*^p < 0.05, ^**^p < 0.005, ^***^p < 0.001). The differences between the MwoA and HC groups are shown in Figure 3.

In the comparison between the VaM and MwoA groups, the VaM group showed significantly stronger activation than the MwoA group in the right angular gyrus, left cuneus, right middle cingulate gyrus, and left precuneus (^*^p < 0.05, ^**^p < 0.005, ^***^p < 0.001). Conversely, the VaM group exhibited weaker activation than the MwoA group in the right middle occipital gyrus (^*^p < 0.05, ^**^p < 0.005, ^***^p < 0.001). The differences in activation between the VaM and MwoA groups are depicted in Figure 3.

## Discussion

This study used visual stimulation combined with fMRI to investigate visual and related brain networks in interictal VaM and MwoA patients compared with HC. The results revealed alterations in visual-related brain networks in both patient groups, with distinct differences. Specifically, compared with HC, the VaM group had stronger activation in the left precuneus. Compared with MwoA, the VaM group showed stronger activation in the right angular gyrus, left cuneus, right middle cingulate gyrus, and left precuneus, but weaker activation in the right middle occipital gyrus. These findings are consistent with current research supporting brain network abnormalities in visual aura migraine (22, 25–30). In recent years, numerous studies have used various imaging techniques to explore the pathogenesis of migraine with aura. Notably, some fMRI studies have observed a pattern of BOLD signal changes during the aura phase characterized by initial hyperperfusion followed by hypoperfusion, spreading in a manner consistent with CSD, providing a mechanistic explanation for visual aura (25). The brain regions showing enhanced activation in our study are key hubs in higher-order networks involved in visuospatial attention, integration of self-referential stimuli, and cognitive control. This suggests that aberrant excitability in higher-order cortices may predispose to CSD. Furthermore, previous research found differences in activation between the symptomatic and asymptomatic hemispheres in interictal VaM patients, as well as compared with controls, involving regions such as the inferior parietal lobule, inferior frontal gyrus, and superior parietal lobule, which encompass various visual-driven functional networks (22) This indicates hyperexcitability in visual centers in migraine, potentially leading to visual aura. Additionally, several studies have reported hyperexcitability and metabolic abnormalities in the visual cortex during the interictal perio (26–30), aligning with our conclusions. However, the early characteristics of visual network changes still require further investigation.

The cuneus, located medial to the calcarine sulcus and connecting the bilateral occipital lobes, participates in processing and integrating visual information and relays it to higher-order cortices. It can respond synchronously with the occipital lobe to visual stimuli (31, 32). The middle occipital gyrus is also a visual processing area responsible for acquiring and inputting visual information and is involved in processing visuospatial information (33). Our study demonstrated that VaM patients had higher activation in the cuneus but lower activation in the middle occipital gyrus compared with MwoA patients. This pattern resembles the increased Cohe-ReHo in the visual network reported during the acute phase of multiple sclerosis (34), suggesting that VaM patients also exhibit visual network abnormalities. The aberrant activation of the cuneus and functional disruption of the occipital lobe may serve as preliminary indicators warranting further investigation into the generation of visual aura. Moreover, our results showed that the MwoA group had significantly higher activation in the middle occipital gyrus than the HC group. This region is involved not only in visual processing but also in pain modulation (35). Its abnormal activation may be related to the central mechanisms of photophobia and the headache attack process, requiring further study.

The precuneus constitutes the posterior medial parietal cortex. It is structurally complex, deeply located, and has extensive connections with other parts of the parietal cortex, frontal motor areas, and occipital cortex. It is considered a core hub of the default mode network (DMN) and is involved in visuospatial and visuomotor integration, facilitating visuospatial imagery (36). Although research on the relationship between the precuneus and visual aura remains relatively limited, our observation of significantly stronger precuneus activation in VaM than in MwoA under visual stimulation—combined with the region‘s structural complexity and its physiological functions related to visual processing—leads us to cautiously suggest that abnormal interictal precuneus activation might be associated with visual aura occurrence. This interpretation requires confirmation in future studies.

The lingual gyrus, situated in the occipital lobe, is structurally and functionally closely linked to the cuneus and participates in visual processing (37). Previous studies have found that damage, altered functional connectivity, and metabolic abnormalities in the lingual gyrus can induce various visual symptoms such as spatial neglect, abnormalities in the horizontal line bisection test, and visual snow. The lingual gyrus is also considered a brain region involved in the initiation and propagation mechanisms of migraine aura (38–41). Our results showed that both VaM and MwoA patients exhibited lingual gyrus and cuneus activation to varying degrees under visual stimulation. Although the between-group difference in lingual gyrus activation did not reach statistical significance in the final analysis (which might be related to difficulty in recruiting VaM patients leading to a relatively small sample size), the activation difference in the cuneus was statistically significant. Nonetheless, the important role of the lingual gyrus in visual processing tasks cannot be denied. We preliminarily speculate that abnormal cuneus activation may be one of the key brain regions involved in visual aura generation.

The cingulate gyrus can be divided into anterior, middle, and posterior parts, each densely connected to different regions and involved in cognition, sensation, movement, and emotional regulation (42, 43) The angular gyrus, part of the parietal lobe, is critically involved in various cognitive functions. It is closely connected to the occipital cortex and is responsible for processing visual language. Studies have shown that migraine patients can experience cognitive impairment both during and between attacks. Recurrent attacks can also lead to anxiety and depressive disorders, particularly evident in patients with chronic or refractory migraine. Although such mood disorders can affect cognition, the cognitive impairment cannot be entirely explained by comorbidities alone (44, 45). In our study, eight VaM patients with a longer disease course had experienced transient anxiety and depression. Concurrently, our results indicated stronger activation in the cingulate gyrus in the VaM group than in the MwoA group. This could be tentatively related to the long-term dual impact of visual aura and headache on these patients, potentially contributing to mood disorders and a greater demand for central regulatory mechanisms; however, this hypothesis remains speculative. The correlation between the cingulate gyrus, angular gyrus, and cognitive dysfunction in migraine patients requires further investigation.

## Limitations

This study has several limitations, which also point to directions for future research. First, Sample size and statistical power: No *a priori* sample size calculation was performed due to the exploratory nature and lack of reliable effect size estimates from prior task-fMRI studies directly comparing VaM, MwoA, and HC under visual stimulation. Post-hoc power analysis based on conventional thresholds (80% power, *α* = 0.05) indicated that detecting a large effect (Cohen’s d ≥ 0.8 for two-group comparisons, corresponding to *f* ≥ 0.4 for three-group ANOVA) would require 26 participants per group for a two-sample t-test and 22 per group for a three-group ANOVA. Our sample of 21 per group is therefore marginally adequate for detecting large effects in two-group comparisons and sufficient for the ANOVA. However, the study is underpowered for medium or smaller effects: detecting a medium effect (d = 0.6, *f* = 0.3) would require 45–50 per group, and detecting a small-to-medium effect (d = 0.5, *f* = 0.25) would require 64–65 per group. This is particularly relevant to our study, where several brain regions showed significant differences at *p* < 0.05 (uncorrected) but with moderate effect sizes, suggesting that these findings may be unstable and require replication. Consequently, the present findings should be interpreted as exploratory, and the absence of significant activation in certain regions should not be taken as evidence of no effect. Replication in larger independent cohorts is essential. Second, statistical thresholding: The primary whole-brain analysis used a liberal voxel-wise threshold of *p* < 0.05 (uncorrected) with a minimal cluster size of 8 voxels. As a supplementary sensitivity analysis, we also applied false discovery rate (FDR) correction at q < 0.05. The core findings—including the left precuneus (VaM vs. HC), left cuneus and right angular gyrus (VaM vs. MwoA)—remained detectable under FDR correction, although the number of suprathreshold voxels was substantially reduced. This consistency suggests that the main results are not purely driven by the liberal threshold. Nevertheless, the uncorrected threshold was retained as the primary exploratory analysis to avoid type II errors (false negatives) given the relatively small sample size. We acknowledge that the lack of family-wise error (FWE) or non-parametric permutation-based correction remains a limitation. Consequently, the spatial specificity and reproducibility of the reported activations should be interpreted with caution, and all findings should be considered exploratory, requiring replication in larger independent cohorts with more rigorous multiple-comparison correction. Third, methodological scope: This study only employed task-state fMRI without integrating other modalities (e.g., resting-state fMRI, MRS, or MEG). Future multimodal investigations are needed to comprehensively characterize the neural mechanisms of visual aura. Fourth, clinical heterogeneity: The visual aura symptoms varied among VaM patients (phosphenes, scintillating scotoma, etc.), but the small sample size precluded subgroup analyses. Future studies with larger samples should examine whether different aura phenotypes are associated with distinct brain activation patterns.

## Conclusion

In summary, our preliminary findings suggest that patients with visual aura migraine (VaM) exhibit abnormal activation patterns across multiple brain regions. This suggests that the generation of visual aura may not be mediated by a single brain area but rather involves a widespread and complex neural network that is functionally aberrant in VaM. However, given the exploratory nature of the analysis and the limitations described above, these findings should be interpreted with caution and validated in independent cohorts using more rigorous statistical approaches.

Specifically, abnormal activation in regions such as the cuneus, precuneus, and superior occipital gyrus may be related to the mechanisms underlying visual aura. Conversely, activation changes in areas like the middle cingulate gyrus and angular gyrus may be associated with cognitive and emotional regulatory dysfunction related to migraine.

Currently, neuroimaging research on migraine pathogenesis has primarily focused on structural imaging and resting-state functional connectivity analysis, while task-state fMRI studies targeting the visual system remain relatively limited. This study employed a visual event-related design, which offers good spatiotemporal resolution, allowing a more direct revelation of visual network activation characteristics. This approach can be extended to in-depth investigations of structural abnormalities within visual-related networks. Our study provides new imaging clues for exploring the central mechanisms of VaM and may offer objective references for its clinical diagnosis and differential diagnosis.

## Data Availability

The original contributions presented in the study are included in the article/supplementary material, further inquiries can be directed to the corresponding authors.
